# Supplementary material for: HAT cofactor TRRAP modulates microtubule dynamics via SP1 signaling to prevent neurodegeneration
Source: eLife. 2021 Feb 17;10:e61531. doi: 10.7554/eLife.61531 (PMC7939550; doi:10.7554/eLife.61531)
Supplement: Source data 1. — (A) Top30 nervous system processes targets of Sp1. DEGs in all three RNA-seq data sets were compared with the list of the Sp1 targets from the Harmonizome database (Rouillard et al., 2016) and the resulting list was analyzed using IPA to find the disease process associated with the DEGs (cutoff, p<0.05). (B) Top30 differentially expressed Sp1 targets. DEGs in the RNA-seq data sets were compared with the list of Sp1 targets from the Harmonizome database and the Top30 DEGs (cutoff, p<0.05) are indicated. (C) Top30 protein changes of Sp1 targets. Proteins from the forebrain, whose expression changed after the Trrap deletion and correlated with the changes in RNA-seq, were compared with the list of Sp1 targets obtained from the Harmonizone database. The Top30 results based on the q-value are summarized. [file elife-61531-data1.docx]

**Source data 1A. Top 30 nervous system processes targets of SP1**

| Pathways | Cortex | Striatum | aNSC |
| --- | --- | --- | --- |
| Cell movement of neurons |  |  |  |
| Neurodegeneration of brain |  |  |  |
| Neurodegeneration |  |  |  |
| Degeneration of CNS |  |  |  |
| Degeneration of NS |  |  |  |
| Proliferation of CNS |  |  |  |
| Proliferation of brain cells |  |  |  |
| Cell movement of brain cells |  |  |  |
| Neurodegeneration of neurites |  |  |  |
| Migration of neurons |  |  |  |
| Neurodegeneration of hippocampus |  |  |  |
| Outgrowth of axons |  |  |  |
| Growth of brain |  |  |  |
| Cell death of cerebral cortex cells |  |  |  |
| Growth of neurites |  |  |  |
| Outgrowth of neurites |  |  |  |
| Cell viability of tumor cell lines |  |  |  |
| Proliferation of neuronal cells |  |  |  |
| Growth of axons |  |  |  |
| Outgrowth of neurons |  |  |  |
| Outgrowth of cells |  |  |  |
| Cerebral cortical atrophy |  |  |  |
| Astrocytosis |  |  |  |
| Gliosis |  |  |  |
| Gliosis of brain |  |  |  |
| Cell death of tumor cell lines |  |  |  |
| Viability of neuroblastoma cell lines |  |  |  |
| Brain atrophy |  |  |  |
| Astrocytosis of brain |  |  |  |
| Synthesis of lipid |  |  |  |

-2,7

2,7

Log_2_ (Fold Change)

**Source data 1B. Top 30 differentially expressed SP1 target genes**

| Genes | Cortex | Striatum |
| --- | --- | --- |
| Gng4 |  |  |
| Flna |  |  |
| Tubb3 |  |  |
| Tmsb10 |  |  |
| Pfkp |  |  |
| Tgfb1i1 |  |  |
| Adam19 |  |  |
| Gap43 |  |  |
| Acsl5 |  |  |
| Slc20a1 |  |  |
| Gng3 |  |  |
| Miat |  |  |
| Hmgcs1 |  |  |
| Actb |  |  |
| Anp32a |  |  |
| Pcbp4 |  |  |
| Arhgdig |  |  |
| Dpysl2 |  |  |
| Uchl1 |  |  |
| **Stmn3** |  |  |
| Plcxd2 |  |  |
| Idh1 |  |  |
| Hmgcr |  |  |
| Tuba1a |  |  |
| Top2a |  |  |
| Lsm11 |  |  |
| Chd1l |  |  |
| Cdc42ep2 |  |  |
| **Stmn4** |  |  |
| Mob3a |  |  |

-2,5

2,5

Log_2_ (Fold Change)

**Source data 1C. Top 30 protein changes of SP1 targets**

1,5

| Rab26 |  |
| --- | --- |
| Syt12 |  |
| **Stmn4** |  |
| Sh2d3c |  |
| Nefh |  |
| Rusc1 |  |
| Pgam2 |  |
| Pcmtd2 |  |
| Nefl |  |
| Nudt15 |  |
| Kpna2 |  |
| Padi2 |  |
| **Stmn2** |  |
| Nefm |  |
| Hmgcs1 |  |
| Fam173a |  |
| Srr |  |
| Sqle |  |
| Gng4 |  |
| Ric8a |  |
| Rasgrp2 |  |
| Olfml3 |  |
| Ptrhd1 |  |
| Phf20l1 |  |
| Atl1 |  |
| Cpped1 |  |
| **Stmn3** |  |
| Nrip3 |  |
| Ccdc184 |  |
| Rtn4 |  |

-1,5

Log_2_ (Fold Change)
